# The effectiveness of multivariate and univariate spectrophotometric techniques for the concurrent estimation of ornidazole and ciprofloxacin HCl in tablet formulation and spiked serum: estimating greenness and whiteness profile

**DOI:** 10.1186/s13065-024-01126-1

**Published:** 2024-01-23

**Authors:** Amir A. Sakur, Duaa Al Zakri

**Affiliations:** Analytical and Food Chemistry Dept., Faculty of Pharmacy, Aleppo University, Aleppo, Syria

**Keywords:** Artificial intelligence, Bayesian Regularization, Fourier self-deconvolution, Green certificate, Levenberg–Marquardt, Mean centering

## Abstract

In this manuscript, the effectiveness of multivariate and univariate tools in conjunction with spectrophotometric techniques was evaluated for the concurrent analysis of ciprofloxacin (CI) and ornidazole (OR) in prepared mixtures, tablets, and human serum. The artificial neural network was chosen as the multivariate Technique. Bayesian regularization (trainbr) and Levenberg–Marquardt algorithms (trainlm), were constructed and trained using feed-forward back-propagation learning. The optimal logarithm was determined based on mean recovery, mean square error of prediction (MSEP), relative root mean square error of prediction (RRMSEP), and bias-corrected MSEP (BCMSEP) scores. Trainbr outperformed trainlm, yielding a mean recovery of 100.05% for CI and 99.84% for OR, making it the preferred algorithm. Fourier self-deconvolution and mean-centering transforms were chosen as the univariate Techniques. Fourier self-deconvolution was applied to the zero-order spectra of ciprofloxacin and ornidazole by electing an appropriate full width at half maximum, enhancing peak resolution at 380.1 nm and 314.2 nm for CI and OR, respectively. Mean centering transform was applied to CI and OR ratio spectra to eliminate constant signals, enabling accurate quantification of CI and OR at 272.0 nm and 306.2 nm, respectively. The introduced approaches were optimized and validated for precise CI and OR analysis, with statistical comparison against the HPLC method revealing no notable differences. The sustainability of these approaches was confirmed through the green certificate (modified eco-scale), AGP, and whiteness-evaluation tool, corroborating their ecological viability.

## Introduction

Vaginal dysbiosis poses a significant challenge in obstetrics and gynecology, owning a prevalence of 10–30% in women worldwide. Vaginal dysbiosis is common over pregnancy and several studies have noted a relation between bacterial vaginosis and premature birth in addition to miscarriage and low birth weight [1, 2]. Recent research highlights the therapeutic potential of a ciprofloxacin HCl and ornidazole combination for managing bacterial vaginosis and vaginal dysplasia associated with mixed anaerobic and aerobic bacterial presence [3]. Another study underscores the clinical efficacy and tolerability of an antimicrobial-antiprotozoal combination comprising ornidazole and ciprofloxacin in postoperative complications, cystitis, and overactive bladder treatment [4].

**Fig. 1:**
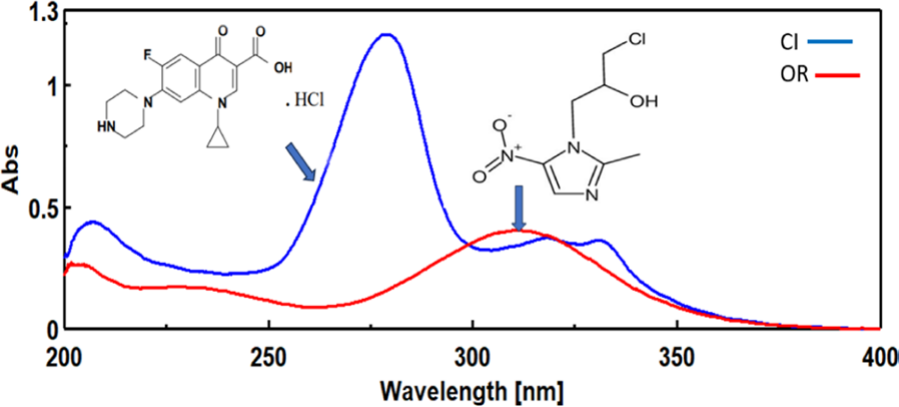
UV spectra and chemical structure of CI and OR

Ornidazole (OR), Fig. 1, exhibits bactericidal activity against anaerobic bacterial infections such as amoebae and trichomonas, disrupting microbial DNA synthesis [5, 6]. Ciprofloxacin HCl (CI), Fig. 1, is a broad-spectrum antibacterial agent effective against respiratory, urinary, and skin infections, inhibiting bacterial DNA replication [7, 8].

Previous literature has reported the estimation of ciprofloxacin and ornidazole combination in tablet form using HPLC [9–12] and spectroscopy [13–16]. Concurrent estimation in spiked serum has been documented using TLC [17] and HPLC [18] only.

Multivariate technique represented by an artificial neural network (ANN) is of significant importance. ANN eliminates the error resulting from employing a single wavelength regression and is therefore robust. Also, the various concentration ratios covered by ANN are highly broad and can include future combinations of ratios created by pharmaceutical corporations. Univariate techniques like Fourier self-deconvolution, and mean centering are distinguished by simplicity in resolving the interfering drugs without requiring complicated mathematical procedures or affecting the signal-to-noise ratio.

This study pioneers the employment of artificial intelligence via two logarithmic methods (trainbr, trainlm), Fourier self-deconvolution, and mean centering in spectrophotometric analysis for concurrent quantification of CI and OR in pharmaceutical formulations and human serum without complex sample preparation or separation. The strengths and limitations of these approaches are debated comprehensively, highlighting their performance. Furthermore, a statistical comparative study was conducted between the introduced approaches and the HPLC approaches to assure efficacy.

In alignment with the role analytical laboratories play in environmental protection by monitoring pollutants, this work embraces the principles of green chemistry, emphasizing operator safety, responsible solvent usage, waste reduction, and energy efficiency [19, 20]. This endeavor thus provides eco-friendly, intelligent, and straightforward methodologies for routine CI and OR analysis.

## Theory

### Artificial neural network (ANN)

**Fig. 2:**
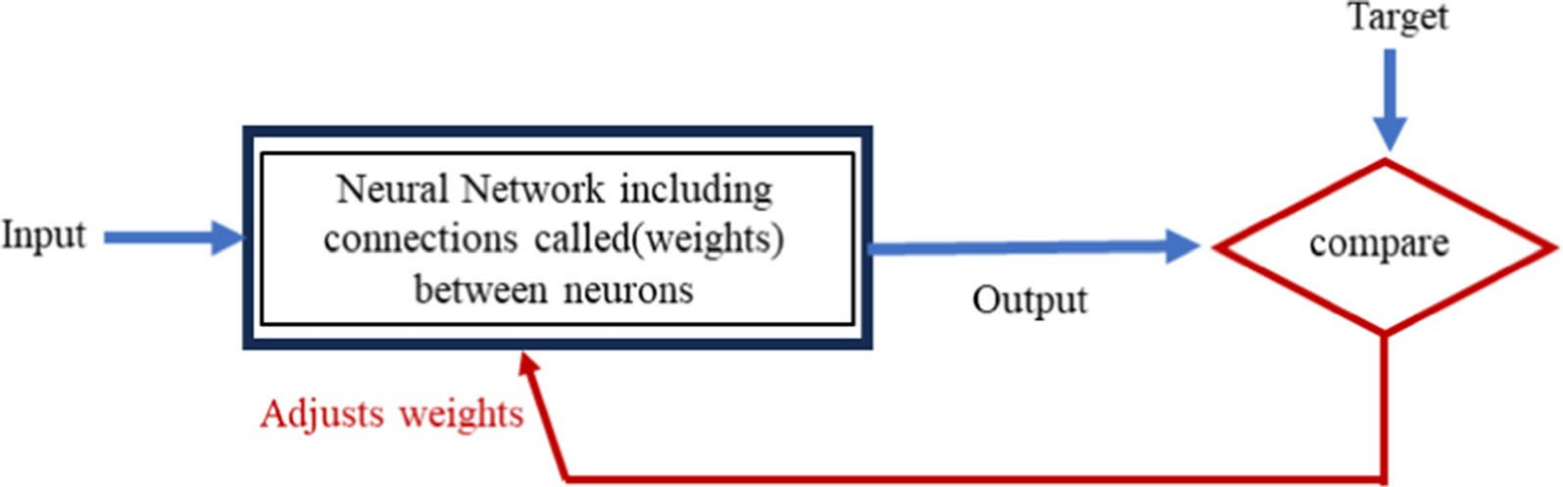
Network training method

An Artificial Neural Network (ANN) is an effective model that simulates the functioning of the human brain's neural networks. Neurons, the basic units of ANN, structurally resemble human nerve cells. Training an ANN involves adjusting its weights and biases to attain a specific objective as illustrated in Fig. 2 [21]. In this study, a feed-forward network trained with backpropagation [22] was used. Different learning algorithms exist for neural networks, including Levenberg–Marquardt backpropagation, Bayesian regularization backpropagation, Resilient backpropagation, and One-step secant backpropagation, among others. For this study, we employed two algorithms: Bayesian regularization backpropagation and Levenberg–Marquardt backpropagation. Bayesian regularization reduces squared errors and weight combinations, ensuring a generalized network. It is robust and does not demand validation [23]. Levenberg–Marquardt, introduced by Donald Marquardt, addresses nonlinear least squares issues and enhances second-order training speed without necessitating the computation of the Hessian matrix, as in Newton's algorithm [24].

### Fourier self-deconvolution (FSD)

This function in the spectra software is an advanced combination of the Fourier self-deconvolution technique (FSD) proposed by Kauppinen et al. and the Finite Impulse Response Operator edited by Jones and Shimokoshi [25]. Given E(ν) as the actual spectrum, M(ν) as the measured spectrum, and G(ν) as the spectrum altered by instrumental measurement, the deconvoluted spectrum is expressed through a Fourier transformation (F) as follows:

$$E\left(v\right)=F\left\{\frac{D(x,L)}{exp\left\{\mathrm{\pi \sigma }\left|x\right|\right\}}\right\}*M(v)$$

Here, D(x, L) is the tapering function, and exp{πσ x} represents the Lorentzian curve's half-width of G(ν).

To optimize computation time, an algorithm that combines the FSD and FIRO techniques is utilized, where the FIRO technique includes extending the exp{ πσ x}expression in the previous equation on the spectrum area for diverse types of tapering functions. The deconvolution filter applied to the spectrum takes the form:

$$F\left\{\frac{D(x,L)}{exp\left\{\mathrm{\pi \sigma }\left|x\right|\right\}}\right\}*D\mathrm{^{\prime}}(v,FL)$$

Here, D(x, L) and D′(v, FL) are constants related to the Bessel function, and L and FL are automatically determined values. The FSD algorithm built within the Spectra Manager software is a powerful technique for resolving overlapping peaks, achieved by applying an appropriate full width at half maximum (FWHM) to the spectrum, producing a deconvoluted spectrum with zero-crossing points (λzero). The concentration of each component is detected then by the constructed linear equation between the signal at λzero and the analogous concentrations.

### Mean centering (MC)

Mean centering is a data transformation technique via MATLAB® designed to handle highly overlapped signals by eliminating redundant signals while preserving variable signals [26].

Considering a vector with 3-dimensional:

$$x=\left[\begin{array}{c}3\\ 6\\ 3\end{array}\right]$$

The previous column is then mean-centered by deducting the average of the three numbers.

Utilizing $$\overline{x }=\left[\begin{array}{c}4\\ 4\\ 4\end{array}\right]$$

MC(x) =  $$x-  \overline  {x} = \left[\begin{array}{c}3\\ 6\\ 3\end{array}\right]-\left[\begin{array}{c}4\\ 4\\ 4\end{array}\right] =\left[\begin{array}{c}-1\\ 2\\ -1\end{array}\right]$$

In a mixture consisting of X and Y components, the absorbance (A) is expressed as:

1 $${\text{AM}}\, = \,{\text{ax Cx}}\, + \,{\text{ay Cy}}$$

Where (a) represents the absorptivity factor, and (C) represents the component’s concentration.

When dividing Eq. (1) by component Y spectrum, we obtain:

2 $${\text{AM}}/{\text{ay}}\, = \,{\text{ax Cx}}/{\text{ay}}\, + \,{\text{Cy}}$$

Utilizing MATLAB®, mean centering is applied to the ratio spectra, resulting in:

3 $${\text{MC}}\left( {{\text{AM}}/{\text{ay}}} \right)\, = \,{\text{MC}}\left( {{\text{ax Cx}}/{\text{ay}}} \right)$$

The constant's mean centering value is zero. Equation (3) illustrates that after mean centering, the mixture signal is solely related to component X.

## Experimental

### Instruments and software

The JASCO V-650 spectrophotometer was employed for D0 spectrum scanning in the range of (200–400) nm. Spectra Manager® software, version 2 by JASCO Corporation, was used for Fourier self-deconvolution. MATLAB® 2021a (version 8.6) was utilized for the ANN and mean-centering approaches.

### Materials and solvent

- Ciprofloxacin HCl and Ornidazole were obtained from Chongqing Chemdad CO., Ltd., CHINA, with purities of 99.35 ± 0.73 and 99.16 ± 0.62, respectively.
- Methanol was procured from Panreac, Spain.
- Cifran-OZ® film-coated tablets, each labeled to contain 500 mg of ornidazole and ciprofloxacin HCl, were manufactured in India by Sun Pharma Laboratories Ltd., with batch no. SXC0964A.

#### Standard solutions

- Stock standard solutions of 1000 μg/ml were prepared for both CI and OR in methanol.
- Working solutions of 50 μg/ml were prepared for both CI and OR in methanol.

### Preparing tablet sample

Ten Cifran-OZ® tablets were weighed accurately, and crushed, and an amount equivalent to 10 mg of each CI and OR was dissolved in a 50 ml standard flask using 20 ml of methanol. The solution was sonicated for 5 min, filtered into a 50 ml standard flask, and topped up with methanol. Appropriate dilution was performed to prepare the sample solution.

### Preparing serum sample

The serum sample was human serum collected from three healthy volunteers, then treated as follows: 0.1 ml of serum sample was mixed with various concentrations of CI and OR in centrifugation tubes. The volume was adjusted to 5 ml using methanol, and the mixture was vortexed for 2 min and then centrifuged at 5000 rpm for 10 min. The centrifuged serum sample was filtered utilizing a 0.22 µm Millipore filter and measured against a produced blank under the aforementioned conditions except for the existence of the drugs.

## Procedures and calibration

A series of methanolic solutions were prepared in the laboratory, spanning concentrations from 2 to 15 μg/ml for CI and 3–25 μg/ml for OR. These solutions were recorded against methanol and saved in the spectra software.

### Artificial neural network (ANN)

For trainbr, thirty-two mixtures containing varying ratios of CI and OR, within the concentration ranges of 2–15 μg/ml for CI and 3–25 μg/ml for OR, were prepared for the training set (Table 1). Additionally, a test set comprising 10 mixtures of CI and OR was prepared to evaluate the trained artificial networks. For trainlm, thirty mixtures of CI and OR were utilized as the training test, 6 mixtures as the validation set, and an additional 6 mixtures as the test set.

**Table 1:** Concentrations of training mixtures in μg/ml

Mix no.	CI μg/ml	OR μg/ml
1	2.0	3.0
2	3.0	5.0
3	5.0	10.0
4	8.0	15.0
5	2.5	10.0
6	2.0	5.0
7	5.0	15.0
8	3.0	3.0
9	5.0	5.0
10	8.0	10.0
11	10.0	3.0
12	15.0	0.0
13	3.0	10.0
14	2.0	10.0
15	3.0	15.0
16	5.0	20.0
17	8.0	25.0
18	3.0	10.0
19	5.0	15.0
20	2.0	20.0
21	3.0	25.0
22	5.0	15.0
23	8.0	15.0
24	10.0	5.0
25	2.0	15.0
26	3.0	10.0
28	5.0	15.0
28	8.0	20.0
29	2.0	25.0
30	3.0	3.0
31	5.0	25.0
32	8.0	3.0

### Fourier self-deconvolution method

The recorded spectra of CI and OR were subjected to Fourier self-deconvolution (FSD) using a full-width half-maximum (FWHM) of 70. A calibration plot was established between the signal values at 280.1 nm for CI and 314.2 nm for OR, corresponding to their respective concentrations.

### Mean centering method

The spectra of CI and OR were divided by the spectra of CI (10 μg/ml) and OR (20 μg/ml), respectively. The resulting ratio spectra were subjected to mean centering. Linear equations were developed between the signals at 272.0 nm for CI and 306.2 nm for OR, and their respective concentrations.

## Results and discussion

Upon analyzing Fig. 1, it is apparent that the D0- spectra associated with CI and OR overlap in a manner that hampers the independent quantification of each compound. As a solution, this study introduces three approaches to permit the accurate quantification of individual compounds. The potential of ANN was explored to resolve the CI and OR overlap within the UV range (279.0–316.0) nm at a 0.5 nm interval. The ANN input consisted of an absorbance matrix (75 × 32), while the output matrix (32 × 2) represents the concentrations for CI and OR. The Fourier Self-Deconvolution (FSD) technique's efficiency was assessed through variations in the Full Width at Half Maximum (FWHM) for CI and OR spectra within the UV range (200.0–400.0) nm using a 0.1 nm interval. Additionally, the Mean Centering (MC) approach was applied to CI and OR ratio spectra within the UV range (240.0–290.0) nm for CI and (260.0–340.0) nm for OR using a 0.1 nm interval.

### ANN

To ensure the correctness of the ANN training process and minimize errors, it was crucial to carefully select noise-free and reliable absorbance values for input. In this context, signal points within the range of (279.0–316.0) nm were chosen as the network's inputs, while other spectral points were disregarded. For the ANN training, two algorithms, trainbr, and trainlm, were employed, and three network configurations were considered for each component, consisting of (1 hidden layer, 1 neuron), (1 hidden layer, 2 neurons), and (2 hidden layers, 2 neurons). Activation functions TANSIG and PURELIN were applied in the hidden and output layers, respectively. The output layer of all networks contained only 2 neurons to accommodate the two compounds in the mixtures. It has been determined that training networks with one hidden layer and two hidden layers are sufficient to address the spectral interference of CI and OR. Further hidden layers could potentially exacerbate the issue of overfitting [27]. The performance of each network was evaluated using various parameters, including mean recovery%, mean square error of prediction (MSEP), relative root mean square error of prediction (RRMSEP), and bias-corrected MSEP (BCMSEP) [28].

The formulas utilized for the aforementioned parameters are as follows:

$$MSEP = \frac{{\sum_{i = 1}^n (c^{\prime} - c)^2 }}{n}$$

$$RRMSEP=\frac{100}{\overline{c}}\sqrt{MSEP }$$

$$BCMSEP = \frac{{\sum_{i = 1}^n (c^{\prime} - c)^2 - \left[ {\sum_{i = 1}^n (c^{\prime} - c)^2 /n} \right]}}{n - 1}$$

Here, c': represents the predicted concentration, c: represents the true concentration, c̅: is the average of true concentrations, and n is the sample number.

**Fig. 3:**
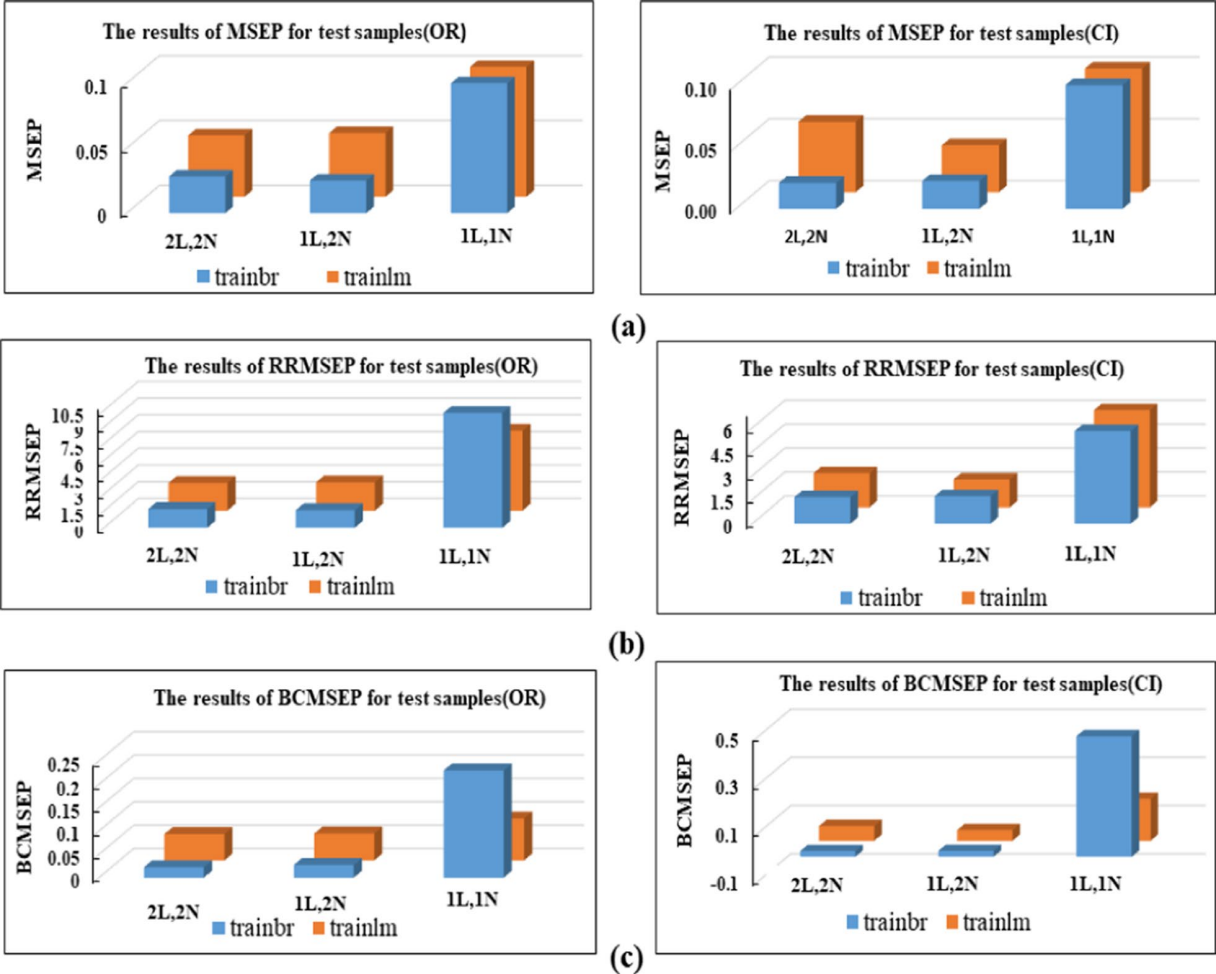
MSEP (**a**), RRMSEP (**b**), and BCMSEP (**c**) results for test samples of CI and OR

**Fig. 4:**
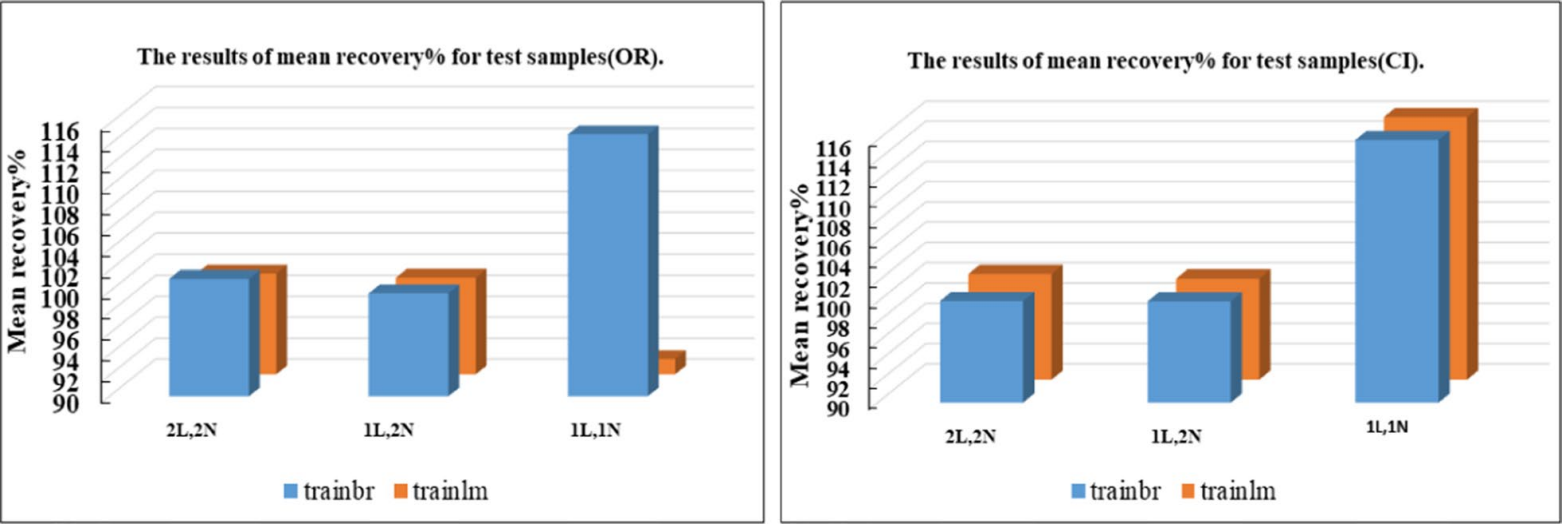
Mean recovery% results for test samples of CI and OR

**Fig. 5:**
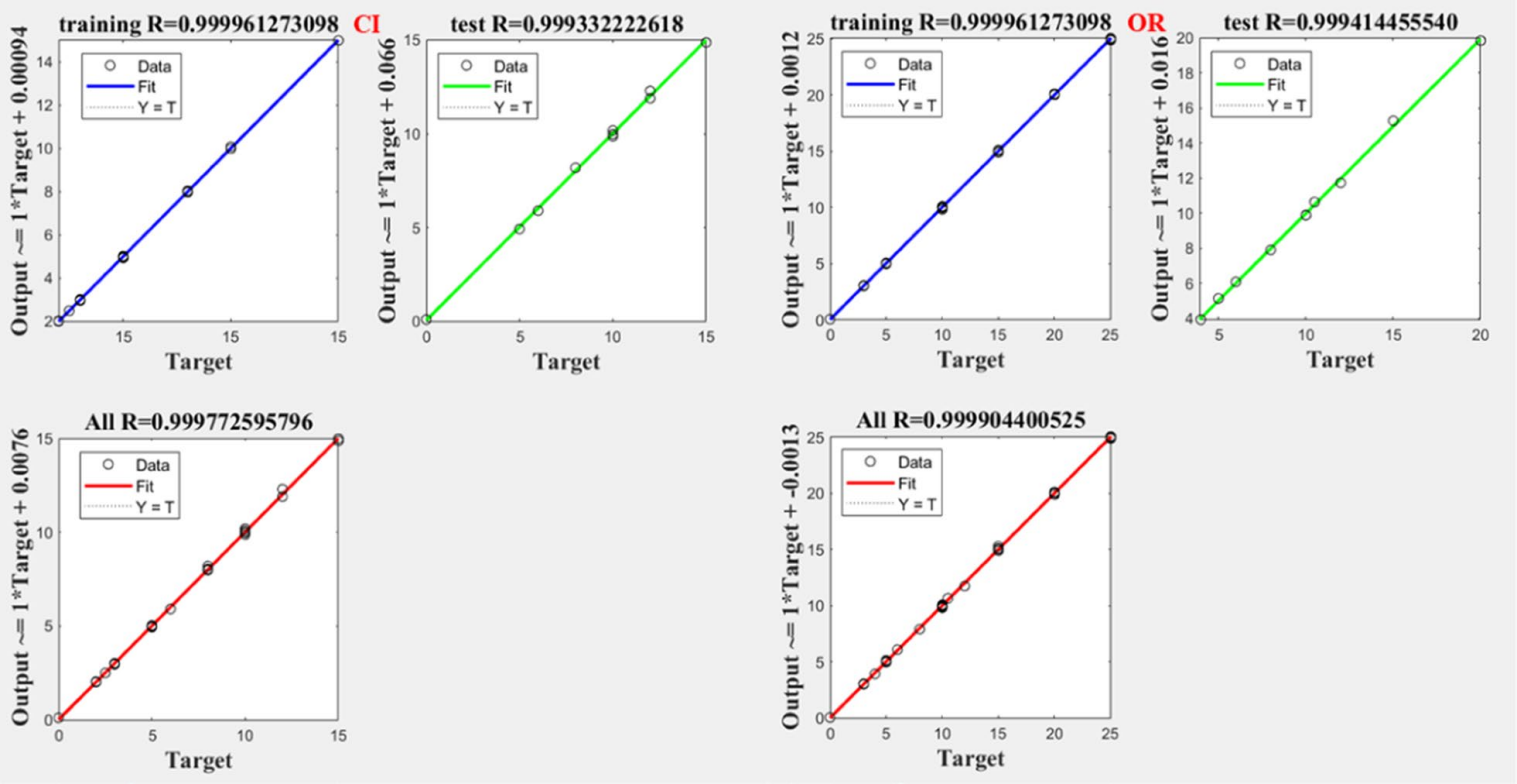
Training, and test diagrams of trainbr (1 layer, 2 neurons) for CI and OR

**Fig. 6:**
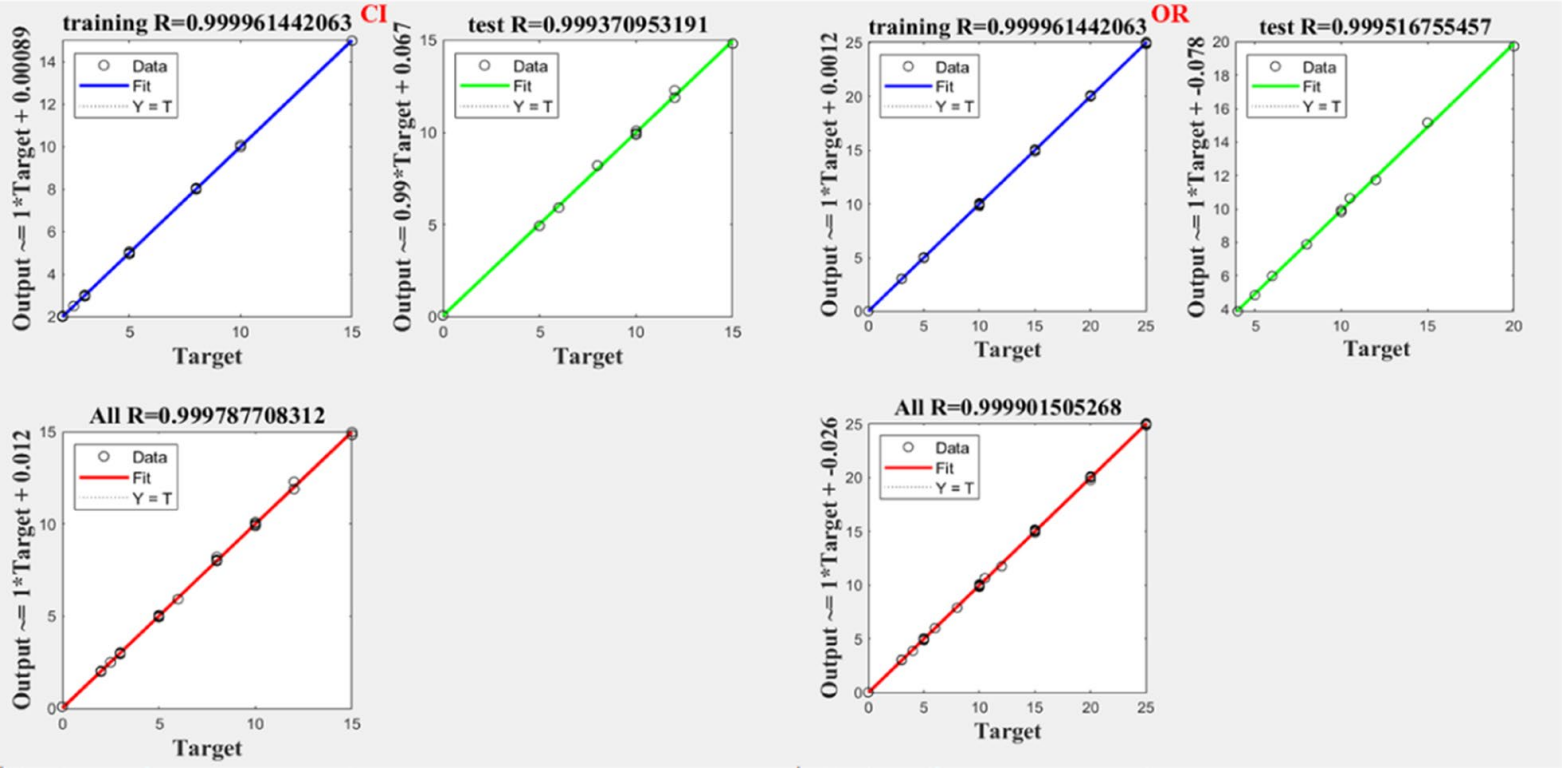
Training, and test diagrams of trainbr (2 layers, 2 neurons) for CI and OR

**Fig. 7:**
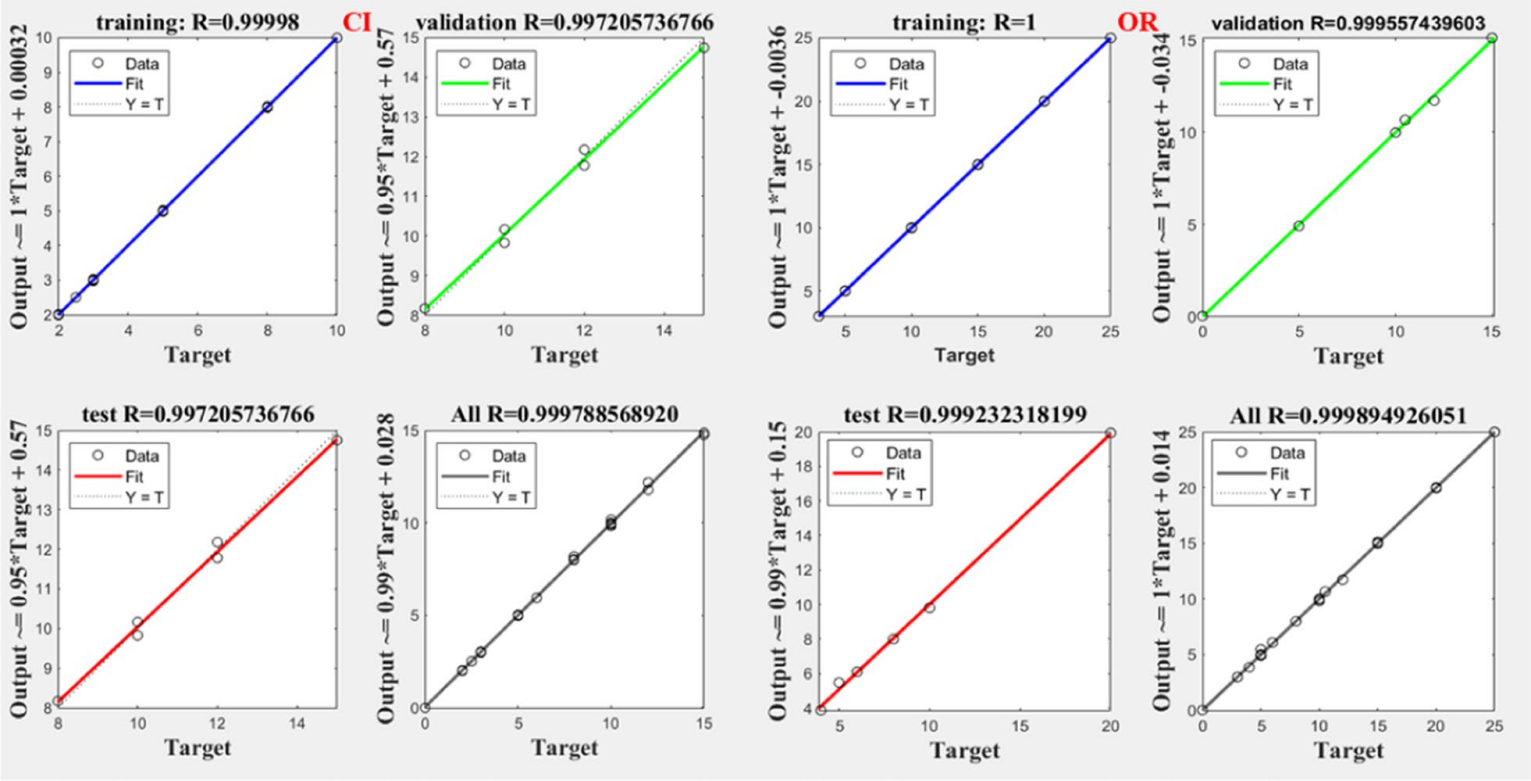
Training, validation, and test diagrams of trainlm (1 layer, 2 neurons) for CI and OR

**Fig. 8:**
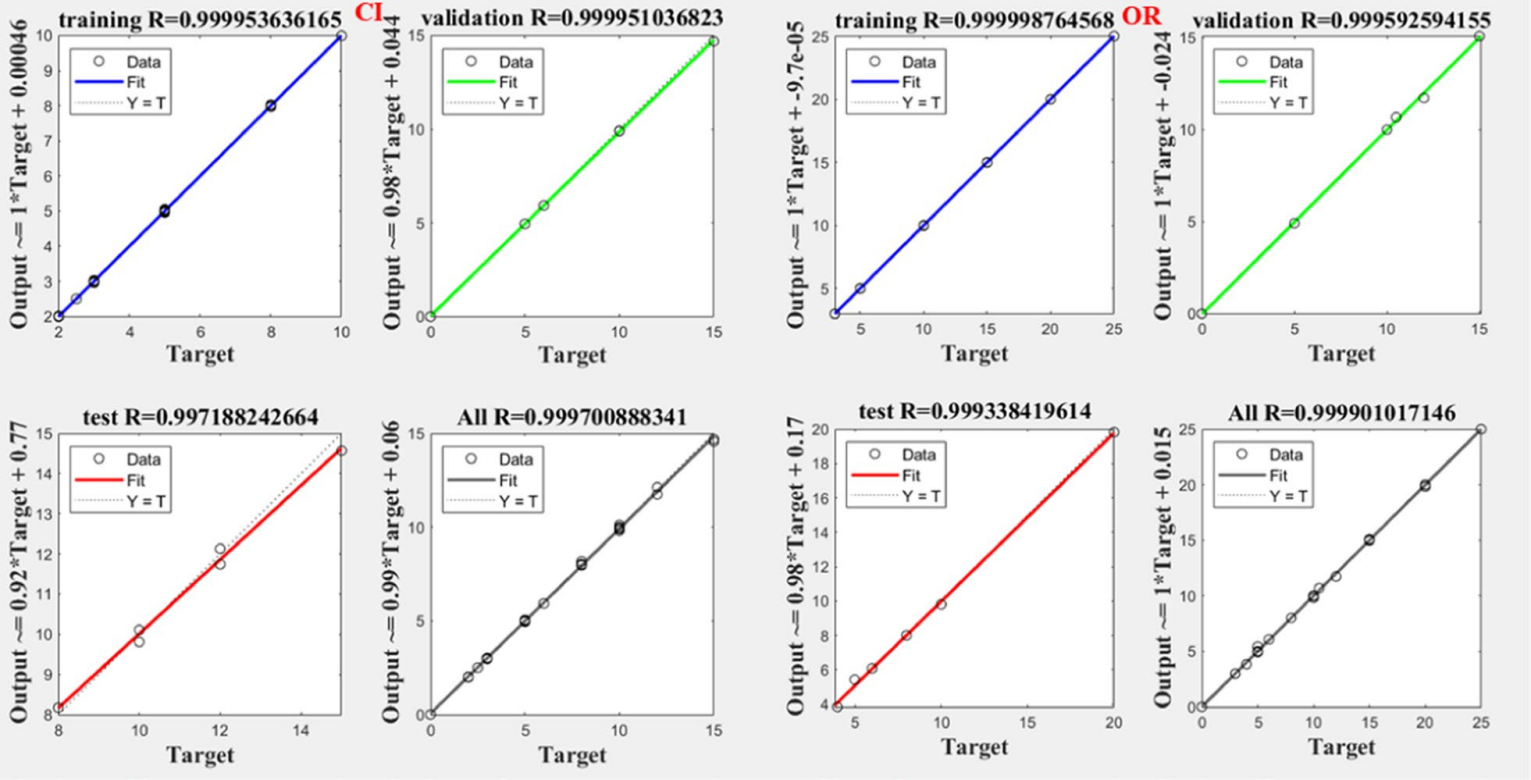
Training, validation, and test diagrams of trainlm (2 layers, 2 neurons) for CI and OR

**Fig. 9:**
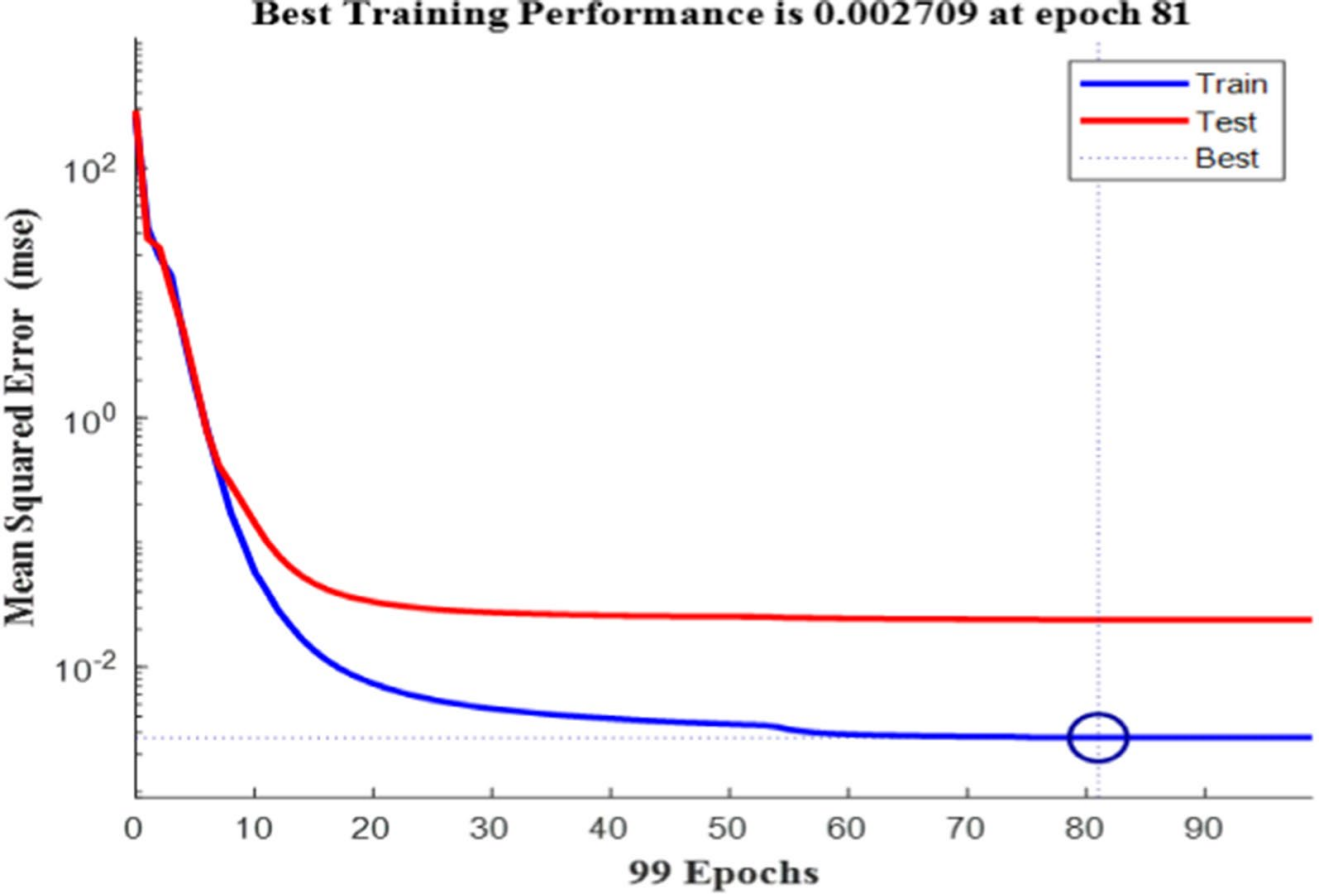
Best training performance of trainbr (1 layer, 2 neurons) for CI and OR predictions in the produced mixes

Notably, all trained networks performed well except the (1 hidden layer, 1 neuron) configuration, which exhibited suboptimal outcomes (Figs. 3, 4). Trainbr demonstrated fewer errors in performance with superior results in terms of MSEP, BCMSEP, and RRMSEP compared to trainlm. Also, as noticed from Figs. 5, 6, 7, 8 the slope value of 1 in trainbr diagrams indicates the excellent performance of the trained net, unlike trainlm, which exhibits a slope value of less than 1 in test diagrams of CI. While there was no notable difference between trainbr with (1 hidden layer, 2 neurons) and trainbr with (2 hidden layer, 2 neurons), the former was chosen for the simultaneous detection of CI and OR in tablet and serum samples. As depicted in Fig. 5 the training and test diagrams of trainbr (1 layer, 2 neurons) for CI and OR reveal an excellent R-value. Furthermore, the mean recovery% and RMSE scores provided in Table 2 validate the algorithm's exceptional performance. These satisfactory results were achieved within 81 epochs, where the gradual decrease of training and test lines confirms the absence of overfitting as indicated in Fig. 9.

**Table 2:** Prediction outcome of test mixes utilizing trainbr (1 layer, 2 neurons)

Test mixture	Real μg/ml		Expected μg/ml		Recovery%	
	CI	OR	CI	OR	CI	OR
1	0.0	10.0	0.01	9.89	–	98.93
2	10.0	15.0	9.99	15.28	99.87	101.84
3	5.0	10.5	4.93	10.64	98.58	101.30
4	6.0	12.0	5.91	11.73	98.47	97.76
5	8.0	8.0	8.19	7.90	102.42	98.70
6	10.0	20.0	10.18	19.86	101.81	99.29
7	12.0	4.0	12.29	3.92	102.41	98.02
8	12.0	6.0	11.89	6.07	99.09	101.25
9	15.0	5.0	14.87	5.13	99.11	102.54
10	10.0	10.0	9.87	9.88	98.73	98.79
Recovery%					100.05	99.84
RMSE					0.073	0.077

### Fourier self-deconvolution (FSD)

**Fig. 10:**
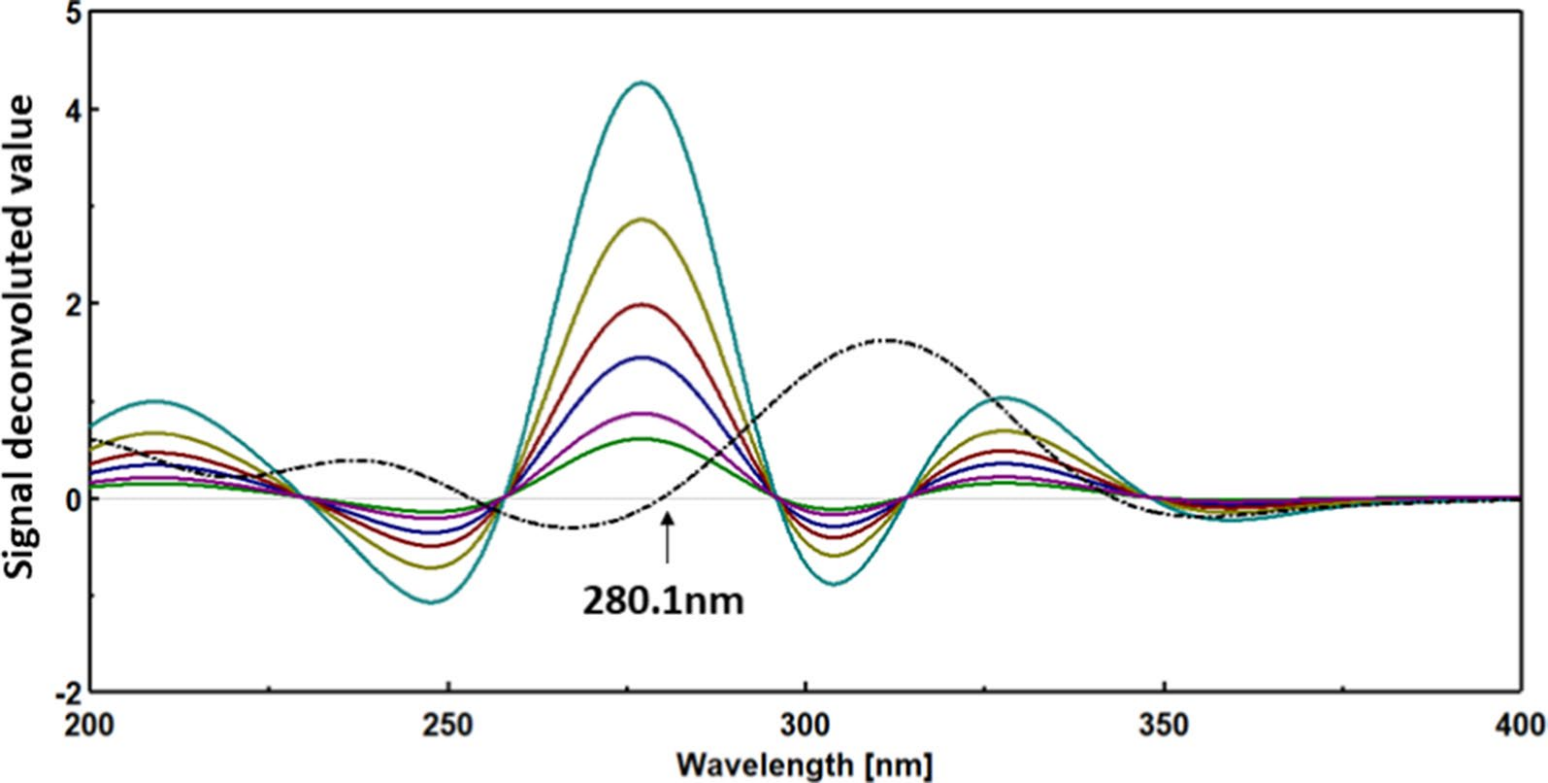
Applying FSD on the UV-spectra of CI(____) 2–15 μg/ml and OR(----) 10 μg/ml using FWHM: 70, displaying zero-crossing point for CI estimation

**Fig. 11:**
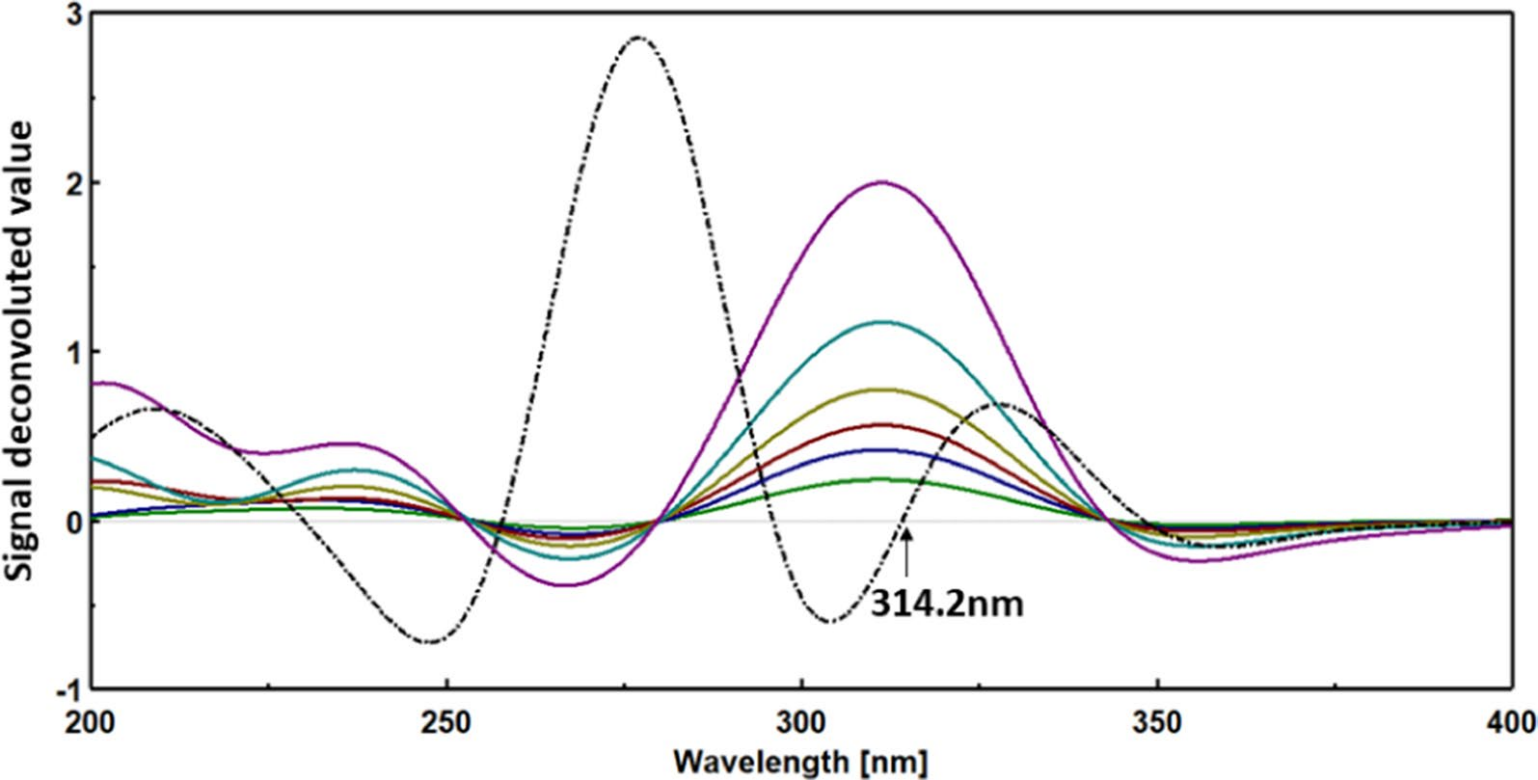
Applying FSD on the UV spectra of OR(____) 3–25 μg/ml and CI(----) 10 μg/ml using FWHM: 70, displaying zero-crossing point for OR estimation

The FSD technique is a significant processing tool that enhances the resolution of overlapping spectra by adjusting the peak widths. This adjustment allows for achieving zero intersection points for each spectrum, thereby enabling the quantification of individual compounds within the drug mixture. Unlike derivative transformation-based methods [29–31], FSD maintains the signal-to-noise ratio and avoids introducing additional noise. To determine the appropriate Full Width at Half Maximum (FWHM) for deconvolution, various FWHM values (10, 30, 50, 70) were applied to the individual spectra of CI and OR within the UV range (200–400) nm using a 0.1 nm interval. The optimal FWHM for deconvolution was determined to be 70, as it allowed for the measurement of each drug without intervention from the other compound. This was achieved at 280.1 nm for CI (Fig. 10) and 314.2 nm for OR (Fig. 11). The following linear equations are utilized to estimate the concentration of each drug at its elected wavelength: for CI Y = 0.2732 X + 0.0031, for OR Y = 0.0778 X + 0.002.

### Mean centering(MC)

**Fig. 12:**
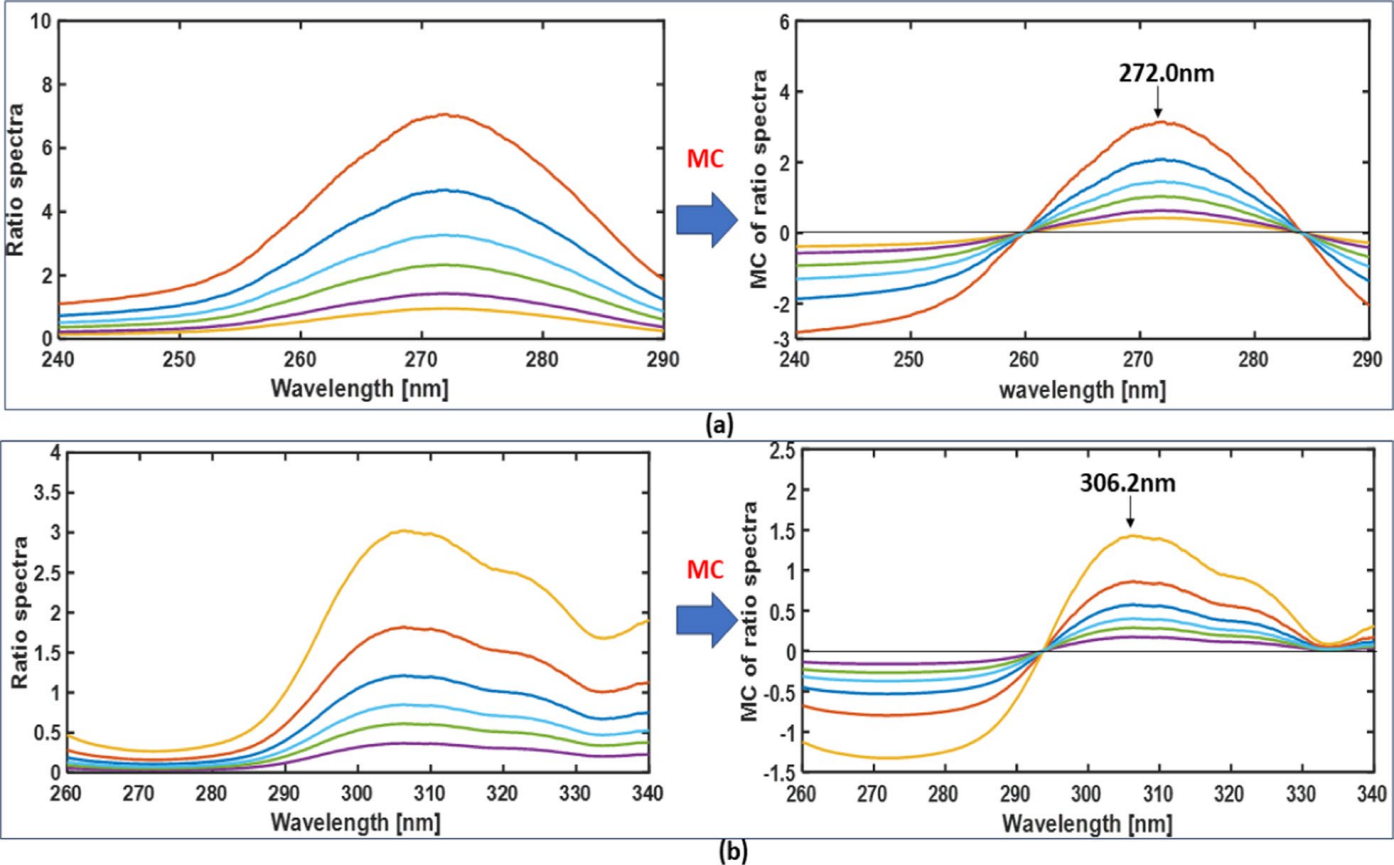
Applying the mean centering function on the ratio spectra of CI (**a**) and OR (**b**)

This tool of data transformation utilizing MATLAB® has the feature of picking the felicitous wavelength for measurement in an expansive range of wavelengths without being restricted to choosing zero points. To apply this method two criteria were tested: the divisor concentration and wavelength range to perform mean centering. The best divisor of CI and OR which accords the best sensitivity and precision were 10 μg/ml of CI and 20 μg/ml of OR, also many wavelength ranges were tried to elect the best range that fulfills the best recovery% scores of CI and OR from the produced mixes as in Table 3, and the elected ranges to accomplish the estimating operation of CI and OR were (240.0–290.0) nm for CI (Fig. 12a) and (260.0–340.0) nm for OR (Fig. 12b). After applying the mean centering function on the produced ratio spectra of CI and OR through the elected wavelength ranges, the λmax at 272.0 nm was elected to detect CI using the following equation Y = 0.2091 X + 0.0026, and the λmax at 306.2 nm was elected to detect OR utilizing the following equation Y = 0.0571 X + 0.0042.

**Table 3:** Effect of wavelength ranges on the recovery results of the prepared mixtures of CI and OR

Wavelength range	Linear equation	Recovery% Drugs concentration (μg/ml) CI: OR		
		10:5	5:15	3:10
CI				
240–290 nma	Y = 0.2091X + 0.0026	99.42	98.92	98.10
230–300 nm	Y = 0.2630X + 0.0030	95.70	96.61	96.41
260–290 nm	Y = 0.1206X + 0.0015	96.07	95.18	92.98
240–325 nm	Y = 0.2943X + 0.0037	95.96	92.08	92.14
250–300 nm	Y = 0.2118X + 0.0028	97.03	95.78	96.82
OR				
260–340 nma	Y = 0.0571X + 0.0042	98.13	99.12	101.09
290–330 nm	Y = 0.0231X + 0.0030	96.27	93.33	97.06
240–340 nm	Y = 0.0609X + 0.0040	104.21	103.94	104.80
260–330 nm	Y = 0.0581X + 0.0041	97.02	97.15	103.06
280–340 nm	Y = 0.0401X + 0.0037	98.37	93.71	97.03

^a^The elected wavelength ranges for mean centering transforms

### Strengths points and limitations of the elaborated tools

Despite the satisfactory and convergent results attained through the elaborating of the three tools for the synchronous quantification of CI and OR, these tools differ from each other by several points that are epitomized in Table 4 to display the strengths points, and limitations of each tool.

**Table 4:** The strengths points and limitations of the elaborated Tools

Tool	Benefits	Limitations
ANN	• Eliminates the error reverting to employing a single wavelength regression such as in univariate UV approaches • Does not demand a monotonous validation process • Can be trained on diverse ratios of mixes	• Needs many tests to obtain accurate prediction outcomes, which needs to adjust many parameters like transfer functions, layers, neuron number, goal, and the learning rate • The improper adjustment of one of the previous parameters may lead to learning errors or the occurrence of overfitting issues
FSD	• Enhances the overlaid spectra resolution • Does not impact the signal/noise ratio • Doesn’t require a special program or (cos, sin) transformation for performing like in Discrete Fourier Transform	• Crucial measurements of the signals at the selected wavelength • Influenced by the increment of wavelength
MC	• The mean-centered signals are measured at the maximum points, for higher sensitivity • Does not impact the signal/noise ratio	• The computed arithmetic mean is greatly affected by skewed data • Needs to test the best divisor concentration and the best wavelength range for the mean centering process

### Confirmation of environmental sustainability

In the current era, the development of analytical methods must prioritize environmental considerations, aiming to uphold ecological balance, minimize waste, reduce energy consumption, and maintain economic viability. Previous research has introduced various tools for evaluating the sustainability of methods. In this study, the sustainability of the proposed approaches was affirmed through the creation of greenness and whiteness profiles using the following methodologies:

#### Green certificate (modified Eco-scale)

**Fig. 13:**
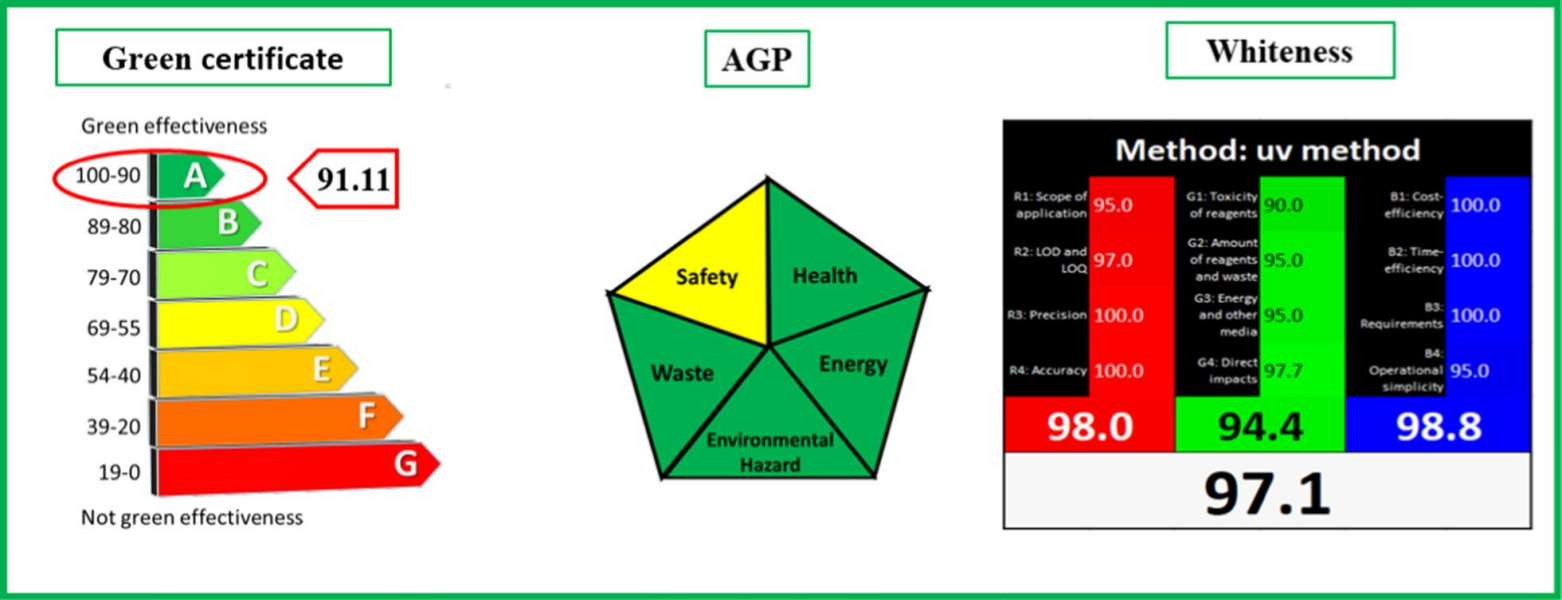
Evaluation of the environmental sustainability of the developed approaches using green certificate, AGP, and whiteness assessment

A new amendment of the eco-scale tool addresses diverse analytical evaluation aspects like reagent volume, hazard, power intake, occupational danger, and generated quantity of wastes in the analysis. This tool relies on a penalty points system and a specific mathematical process to classify the analytical approaches into seven classes according to the final penalty points score; Fig. 13. The penalty points of utilized solvent volume and generated waste are estimated via the following equation:

$${\text{y}}={\text{a}}\times {{\text{x}}}^{{\text{b}}}$$

where a = 0.61 ± 0.05, b = 0.31 ± 0.02 for solvent consumption and a = 1.50 ± 0.08, b = 0.40 ± 0.02 for produced waste. The obtained penalty points for solvent consumption should be multiplied by the penalty points of hazard [32].

#### AGP Tool

This tool evaluates the ecological impact of the procedure based on five criteria: health impact, procedural safety, energy consumption, waste production, and ecological hazards. The evaluation is represented through a pentagram divided into five segments, as illustrated in Fig. 13. The color-coded segments, ranging from green to yellow and red, indicate the varying degrees of ecological impact–high, moderate, and low. Health and safety impacts are estimated based on the red and blue segments of the solvent’s NFPA pictogram, respectively. Waste and ecological hazards are evaluated by considering generated waste during the operation, while energy consumption is estimated based on device usage [33].

#### Whiteness profile

The whiteness profile of the tools was assessed using the RGB 12 algorithm. This method involved populating an Excel template worksheet that comprises three distinct tables: red, green, and blue. Each table corresponds to specific criteria. The green table summarizes factors such as trueness, precision, detection, and quantification limit. The red table focuses on safety, toxicity, and energy consumption, while the blue table addresses cost, speed, and simplicity. The amalgamation of these colors produces white, symbolizing the extent of brightness associated with the method, as illustrated in Fig. 13. By assigning a score from 0 to 100 to each criterion in the Table (0 representing the worst result and 100 signifying a well-fitted method), the final whiteness score, ranging from 0 to 100, is automatically generated [34].

## Validation

### Validation of ANN

The self-validating nature of ANN is a key advantage, and several parameters were computed to assess prediction accuracy [28]. Prediction accuracy was evaluated via MSEP and RRMSEP, while prediction precision was evaluated using BCMSEP. The computed parameters yielded exceptional scores, as summarized in Table 5.

**Table 5:** Construction and Validation Parameters of the ANN approach

Drug	CI	OR
Net	1 layer, 2 neurons	
Transfer functions	TANSIG-PURELIN	
Algorithms	Bayesian regularization	
Regression equation	Y = 1 X + 0.00094	Y = 1 X + 0.0012
MSEPa	0.02257	0.02517
RRMSEP^a^	1.70743	1.57856
BCMSEP^a^	0.02451	0.02749

^a^Calculated for CI and OR at concentrations of (5 µg/mL, 6 µg/mL, 8 µg/mL, 10 µg/mL, 12 µg/mL, 15 µg/mL)

### Validation of fourier self-deconvolution and mean centering methods

The validation of the FSD and MC approaches adhered to ICH guidelines [35] and encompassed the following aspects:

#### Linearity

Calibration plots were established for both CI and OR by plotting the signal against their corresponding concentrations. Remarkable linearity was achieved with an R2 value exceeding 0.999, as detailed in Table 6.

**Table 6:** The validation outcome and parameters of the established tools for CI and OR quantification

Tool	FSD		MC	
Drug substance	CI	OR	CI	OR
Linearity range μg/ml	2–15 μg/ml	3–25 μg/ml	2–15 μg/ml	3–25 μg/ml
Wavelength nm	280.1 nm	314.2 nm	272.0 nm	306.2 nm
Linear equation	Y = 0.2732 X + 0.0031	Y = 0.0778 X + 0.002	Y = 0.2091 X + 0.0026	Y = 0.0571 X + 0.0042
R2	0.9999	0.9998	1	0.9999
Mean% ± SD^a^	100.63 ± 1.23	100.50 ± 1.02	100.20 ± 1.35	100.11 ± 0.83
Repeatability^b^	0.94	0.91	1.10	1.41
Intermediate Precision^b^	1.10	1.75	1.02	1.45
DL μg/ml	0.107	0.163	0.139	0.209
QL μg/ml	0.324	0.495	0.422	0.634

^a^The accuracy of 3 concentrations of CI (4 μg/ml, 6 μg/ml, and 12 μg/ml) and OR (6 μg/ml, 12 μg/ml, and 20 μg/ml)

^b^Intermediate Precision and Repeatability are computed as the RSD% of 3 concentrations of CI (5 μg/ml, 7 μg/ml, and 13 μg/ml) and OR (5 μg/ml, 7 μg/ml, and 15 μg/ml)

#### Detection limit (DL) and quantitation limit (QL).

DL and QL were computed as described in ICH guidelines, utilizing the slope of the calibration plot and the standard deviation of the response. Exceptional DL and QL values underscored the approaches’ sensitivity.

#### Accurac

Accuracy was assessed by computing the mean recovery % for three analyzed concentrations of pure CI and OR, employing their corresponding linear equations. Accuracy was further ensured in commercial formulations utilizing the technique of standard additions. The recovery values, inserted in Tables 6, 8, demonstrated the approaches’ accuracy.

#### Precision

Intraday and inter-day precision were evaluated by computing the RSD% for three analyzed concentrations of pure CI and OR on a single day and over three days, respectively. RSD% values below 2 indicated excellent precision, as detailed in Table 6.

#### Specificity

Diverse mixtures with varying OR and CI ratios, as well as tablet samples, were subjected to analysis via the FSD and MC approaches. The recovery reported in Tables 7, 8 substantiated the excellent specificity of the approaches for estimating CI and OR concentrations in both in-lab mixtures and tablet samples.

**Table 7:** Quantification outcome of CI and OR by the suggested tools in the produced mixes

Tool	FSD		MC	
Drugs concentration (μg/ml) CI: OR	CI	OR	CI	OR
12:4^a^	98.92	100.19	98.81	100.93
12:6	100.10	101.39	99.95	100.80
6:12	98.43	101.85	98.01	101.46
8:8^b^	100.20	100.08	99.17	99.62
Average% ± SD	99.66 ± 1.25	100.88 ± 1.19	98.98 ± 1.08	100.70 ± 1.34

^a^Average of 3 repetitions

^b^A ratio that mimics the announced ratio in tablet formulation

**Table 8:** Quantification outcome of CI, and OR via the suggested tools in tablet formulation and enriched serum

Tablet formulation						
Tool	ANN		FSD		MC	
Drug	CI	OR	CI	OR	CI	OR
Dosage/Tab μg/ml	500	500	500	500	500	500
Assay μg/ml^a^	500.75	500.15	501.25	500.20	496.55	496.95
Recovery% ± SD	100.15 ± 1.67	100.03 ± 1.78	100.25 ± 1.04	100.04 ± 1.15	99.31 ± 1.66	99.39 ± 1.81
Standard addition ^b.c^	99.40 ± 1.88	99.74 ± 1.82	99.98 ± 1.59	99.85 ± 0.24	9.60 ± 0.78	99.65 ± 0.22

Serum sample						
Tool	ANN		FSD		MC	
Drugs ratio(μg/ml) CI: OR	CI	OR	CI	OR	CI	OR
2.6:10.5^d^	98.52 ± 0.85	97.9 ± 1.19	95.37 ± 1.10	98.00 ± 0.33	96.41 ± 1.04	97.39 ± 0.39
10:5	96.42 ± 0.69	95.82 ± 1.50	95.50 ± 0.69	97.40 ± 1.52	95.57 ± 0.68	96.73 ± 1.41
5:15	100.30 ± 1.04	100.26 ± 1.12	96.78 ± 1.04	98.13 ± 0.50	96.13 ± 1.50	97.90 ± 0.14

^a^Average μg/ml for the 3 repetitions of CI and OR

^b^The standard addition is(4,5, 6 μg/ml) for CIPH and ORNI

^c^The computed value is the average % ± SD for 3 concentrations of the standard added with 3 repetitions

^d^Recovery% ± SD for the 3 repetitions of CI and OR

### Statistical evaluation

Statistical comparisons were conducted among the proposed approaches, as well as against the reported HPLC method. T-test, F-test, and One-way ANOVA were employed for this purpose. The scores indicated no notable differences between the proposed approaches and the reported HPLC approach, as summarized in Tables 9, 10. The statistical analyses were performed utilizing the data analysis function in Excel 2019.

**Table 9:** Statistical comparison between the elaborated UV tools and the reference HPLC tool for CI and OR estimation in tablet forms

Tablet formulation	CI			OR			HPLC^a^	
	ANN	FSD	MC	ANN	FSD	MC	CI	OR
Average^b^	100.16	100.57	100.07	100.86	100.53	99.82	100.45	100.28
SD	1.82	0.86	1.59	1.70	1.08	1.49	0.94	1.45
Variance	3.3282	0.7378	2.5243	2.8826	1.1607	2.2267	0.8884	2.1019
t-value^c^	0.3169	0.2034	0.4604	0.5862	0.3082	0.4935	–	–
f-value^c^	3.7464	1.2041	2.8415	1.3715	1.8109	1.0594	–	–

^a^C-18 column utilizing acetonitrile: water as the mobile phase with (45:55v/v) ratio, pH sets to 3.0 via O-phosphoric acid, flow rate 1 ml/min, estimation at 299 nm

^b^n = 5

^c^f(0.05)6.388, t(0.05)2.306

**Table 10:** ANOVA (single factor) outcome for comparing the introduced procedures’ results and HPLC procedures’ results for estimating CI and OR in tablet formulation

Source of variation	Sum of squares	DF	Mean square	F value	P-value	F crit
CI						
Between gro	0.828673	3	0.276224	0.14774	0.929628	3.238872
Within gro	29.91456	16	1.86966			
Total	30.74324	19				
OR						
Between gro	2.901019	3	0.967006	0.462027	0.712699	3.238872
Within gro	33.48746	16	2.092966			
Total	36.38848	19				

### Application on tablet and serum samples

The established ANN, FSD, and MC approaches were applied to tablet formulations and serum samples to predict the actual concentrations of CI and OR. The high linearity range of the elaborated approaches permitted the estimation of CI and OR in human serum, where Cmax = 2.6 µg/mL for CI [36] and Cmax = 10.5 µg/mL for OR [37]. Mean recovery percentages and RSD values were computed for all samples, as presented in Table 8, thereby highlighting the exceptional performance of the proposed approaches in the estimation process.

## Conclusion

In this study, the Bayesian Regularization Network, Fourier Self-Deconvolution, and Mean-Centering transformations have demonstrated their remarkable efficacy for the concurrent quantification of CI and OR in tablet formulations and serum samples. For the multivariate technique, the Bayesian regularization model was elected over Levenberg due to its superior performance and accurate prediction across various drug ratio mixtures. A Bayesian network with (1 layer, 2 neurons) configuration was chosen for the concurrent estimation of ciprofloxacin and ornidazole with excellent scores of MSEP, RRMSEP, BCMSEP, and mean recovery. By employing univariate techniques, FSD with FMHW:70 and MC in the range of (240.0–290.0) nm for CI and (260.0–340.0) nm for OR were both successful in resolving CI and OR overlapping while maintaining the signal-to-noise ratio and exhibiting exceptional sensitivity for the overlapped components. The confirmed sustainability of these methodologies through green certificate, AGP, and whiteness metrics underscores their suitability for routine analysis. Notably, these approaches eliminate the necessity for prior separation processes in drug determination, offering simplicity, speed, and eco-friendliness.

## Data Availability

The data used in this study are available from the corresponding author on rational request.

## Abbreviations

ANN: Artificial neural networks.
trainlm: Levenberg–Marquardt algorithms.
trainbr: Bayesian Regularization.
CI: Ciprofloxacin.
OR: Ornidazole.
FSD: Fourier Self-Deconvolution.
MC: Mean Centering.
AGP: Assessment of Green Profile
